# Impact of germination and kilning parameters on *Eleusine coracana* malting for industrial brewing applications

**DOI:** 10.1038/s41598-025-28926-2

**Published:** 2025-12-29

**Authors:** Teklebirhan Megersa Belihu, Agimassie Agazie Abera, Ermias Abelneh Tesema, Reddy Prasad D M

**Affiliations:** 1 Food and Beverage Industry Research and Development Center, Addis Ababa, Ethiopia; 2 Faculty of Chemical and Food Engineering, Bahir Dar institute of Technology, Bahir Dar University, P. O. Box: 26, Bahir Dar, Ethiopia; 3 Food and Nutrition Research Center, Bahir Dar Institute of Technology, Bahir Dar University, Bahir Dar, Ethiopia; 4 College of Engineering and Technology, Mattu university, Mattu, Ethiopia; 5 Chemical and Energy Engineering Programme Area, Faculty of Engineering, Universiti Teknologi Brunei, Tungku Highway, Gadong, BE1410 Brunei

**Keywords:** Finger millet malting, Eleusine coracana, Germination kinetics, Malt quality, Wort analysis, Brewing quality parameters, Biotechnology, Plant sciences

## Abstract

This study examined the influence of malting conditions on the quality of finger millet (Eleusine coracana, Mecha variety) malt and its brewing potential. A 3 × 3 × 3 factorial design was applied, varying germination temperature (20–30 °C), germination time (2–4 days), and curing temperature (70–90 °C). The malts were characterized by germination energy, enzyme activity, protein and tannin content, as well as brewing performance indicators including diastatic power and hot water extract. Through the interaction of 30 °C germination temperature and 4-day duration meeting international malting standards. Higher germination temperatures and longer durations improved enzyme activity and modification, though accompanied by greater weight loss (20.3–24.5%) and reduced moisture (2.1–4.5%). Wort analysis yielded hot water extract values of 77.7–90.7% (dry basis), with upper values surpassing EBC standards, suggesting possible over-modification under certain treatments. These findings demonstrate finger millet’s suitability as an alternative brewing raw material and support its potential for commercial millet-based beer production in Ethiopia and beyond.

**Supplementary Information:**

The online version contains supplementary material available at 10.1038/s41598-025-28926-2.

## Introduction

Finger millet (*Eleusine coracana*) also known as Dagusa, is one of the important millets believed to be originated in Ethiopia^1^. Around 45% of finger millet is globally produced in Africa, with India being the largest producer (~ 1.8 million tons) and Africa producing approximately 2 million tons annually (FAOSTAT, 2023)^2^. Ethiopia is one of the leading African countries in finger millet production, producing approximately 1 million tons in 2022 and ranking among the top three African producers (FAOSTAT, 2023). It is among the six most important cultivated cereal crops after teff, wheat, maize, sorghum, and barley, based on production area and economic significance (CSA, 2023)^3^.

The processing of finger millet grains into products consists of both traditional and modern methods. Through traditional methods, value-added products such as soaked, cooked, malted, papad, fermented, popped, or puffed, extruded, and multi-grain flour are produced^4^. In Southern Africa the brown cultivar of finger millet is utilized for brewing traditional opaque beer^2^. In Ethiopia, finger millet is used as the most important ingredient to make a traditional opaque beer called tella and a spirit called katicalla/areke. In modern methods, it has great potential to be used in brewing commercial beer^5^. It is used to produce lager beer, though this results in distinctive sensory characteristics including color darkening, yeasty aroma, and raw grain flavor that may require optimization for commercial acceptance^6^. Finger millet beer exhibits higher foam stability than beer produced with sorghum and barley malts due to its high tannin content, though excessive levels may contribute to astringency and require careful process control^7^. Thus, it can serve as a partial substitute for barley in commercial beer production, particularly when supplemented to address lower diastatic power limitations^8^.

In beer production, malting is a controlled forced germination, performed to acquire specific amounts of proteolytic and amylolytic enzymes necessary for brewing applications^9^. Finger millet exhibited good malting qualities with considerable industrial potential without gibberellic acid^10^. The principal challenges to the use of finger millet malt in beer production consist of its small size (which hinders water absorption in steeping), technological and sensory issues, and low diastatic power (DP) as compared to barley and sorghum^7^.

Malting makes essential contributions to beer’s organoleptic characteristics, serving as the primary source of color development and a major contributor to flavor formation^11^. The chemical components in the finger millet malt including sugar, lipids, amino acids, and polyphenols serve as precursors of the color, flavor, and off-flavor compounds^12^. During malting, the enzymatic degradation of polymers is technologically controlled by the degree of steeping, germination time, germination temperature, and kilning/curing conditions^13^.

Temperature affects germination through three primary mechanisms: moisture regulation, hormone synthesis modulation, and enzyme activity optimization^14^. Excessively high temperatures may increase evaporation and decrease moisture content, which would negatively affect germination efficiency and grain viability. In general, the increasing temperature has been found to increase the total germination percentage and germination rate, but exposure to any temperature beyond the optimum range for germination can negatively affect germination^15^. To reduce quality defects of malt, the effect of germination temperature, and curing temperature need to be optimized. This research presents the first comprehensive factorial optimization study examining three-way interactions between germination temperature, duration, and kilning parameters on finger millet (*Eleusine coracana* cv. Mecha) malting kinetics. Our objective was to establish standardized bioprocessing protocols for industrial brewing applications while optimizing multiple quality parameters simultaneously.

## Materials and methods

### Sample collection, transportation and Preparation

The finger millet sample (Mecha variety) was collected from the Adet Agricultural Research Center and then transported to Bahir Dar University, Institute of Technology, Department of Food Engineering Laboratories. The grains were manually cleaned to remove dust, dirt, foreign matter, undersized, broken, and immature grains. Then the sample was stored at room temperature for three weeks to overcome the dormancy.

### Processing

#### Malting process

The malting process involves steeping, germination, and kilning of cereal grains^16^.

##### Steeping

Steeping was done using the modified method of Yenasew (2022)^17^. Finger millet samples (1.5 kg) were weighed, washed 4 times with tap water, and steeped in nylon bags with enough tap water (in a 1:3 grain to water ratio). The steeping was done for 10 h, followed by 2 h aeration, 8 h steeping 2 h aeration, 8 h steeping 2 h aeration, and finally, 4 h steeping (a total of 36 h.) at room temperature to reach an equilibrium moisture content (ca. 45%.). Aeration was done by turning the grain within thirty-minute intervals to allow sufficient air circulation.

##### Germination

The steeped finger millet samples were spread in nylon bags and placed in an incubator (Thermo scientific, model 3311, UK) and were allowed to germinate at about 100% relative humidity. The germination was done at 20^*°*^C, 25^*°*^C, and 30^*°*^C for 2, 3, and 4 days. The germinating grains were turned at 6 h intervals to avoid meshing of the roots and shoots. Water (20 mL per 1.5 kg sample batch) was sprayed using a hand sprayer four times per day to maintain the moisture content and relative humidity during germination.

##### Kilning

The germinated samples underwent controlled kilning in a temperature-regulated drying oven following established kilning temperature and duration protocols based on modified standard methodologies^18^. The samples were kilned at 50^*°*^C for 8 h followed by a 30 min ramp rate to 65^*°*^C. Then, the temperature was adjusted to 65^*°*^C and the sample was dried for 12 h. After kilning, the curing was done according to with little modification^18^. The kilned samples were distributed with the same thickness on aluminum foil and put in the drying oven. Then the kilned samples were cured at 70, 80, and 90^*°*^C using 3 h time to develop color and flavor compounds while maintaining malt quality and temperature-controlled drying oven (AOAC, 2023) for 3 h. The cured sample was cleaned and packed in polyethylene bag to avoid moisture absorbance.

### Determination of grain physicochemical and biological tests

#### Physical properties

##### Thousand kernel weight (TKW)

TKW was determined using the method followed by Bejiga et al., 2020^19^. From the selected and prepared finger millet grains 500 kernels were counted, and weighted then calculated using Eq. (1)

1 $$\:\mathrm{G}=\frac{\mathrm{W}\mathrm{*}1000\mathrm{*}\mathrm{D}.\mathrm{M}}{\mathrm{N}\mathrm{*}100}$$

Where.

G = Weight of thousands of kernels of dry finger millet in grams.

W = weight of 500 kernels taken in grams.

D.M= (100-M), M = Moisture.

N **=** Number of kernels in lot taken.

##### Hectoliter weight (HLW)

HLW was determined according to (AACC) (2022) method 55–10 and the values were adjusted to the moisture content of 12.5% by Eq. (2)^20^.

2 $$\:\mathrm{H}\mathrm{L}\mathrm{W}\:12.5\mathrm{\%}\:\mathrm{M}\mathrm{b}\mathrm{a}\mathrm{s}\mathrm{i}\mathrm{s}=\mathrm{H}\mathrm{L}\mathrm{W}\left(\frac{100-\mathrm{\%}\:\mathrm{m}\mathrm{o}\mathrm{i}\mathrm{s}\mathrm{t}\mathrm{u}\mathrm{r}\mathrm{e}\:\mathrm{m}\mathrm{e}\mathrm{a}\mathrm{s}\mathrm{u}\mathrm{r}\mathrm{e}\mathrm{d}\:\mathrm{i}\mathrm{n}\:\mathrm{g}\mathrm{r}\mathrm{a}\mathrm{i}\mathrm{n}}{100-12.5}\right)$$

#### Biological properties

##### Germination energy (GE)

Germination energy determination followed the modified protocol described by Meng et al. (2017)^21^. Finger millet kernels (100 per replicate) were distributed on moistened filter paper (4 mL distilled water) within 90 mm Petri dishes and incubated under controlled environmental conditions. Germination proceeded at nearly 100% relative humidity across three temperature regimes (20 °C, 25 °C, and 30 °C) for durations of 2, 3, and 4 days. Germination percentage was calculated by counting total germinated kernels at the conclusion of each incubation period.

##### Water sensitivity

This was done as described in the ASBC (2024) Method and cited by Castro et al., 2022^22^.with little modification. Kernels (100) of the sample were placed and uniformly distributed in four Petri dishes lined with two filter papers into two of the dishes, labeled A and B, with 4 mL of water, and into the remaining two dishes, labeled C and D, 8 mL of water was added. All dishes were covered with lids, sealed into a polyethylene bag, and incubated at three different temperatures (20^*°*^C, 25^*°*^C, &30^*°*^C). Then the chitted kernels were removed and counted after 72 h and the water sensitivity (WS) was calculated using Eq. (3)

3 $$\:\mathrm{\%}\mathrm{W}\mathrm{a}\mathrm{t}\mathrm{e}\mathrm{r}\:\mathrm{s}\mathrm{e}\mathrm{n}\mathrm{s}\mathrm{i}\mathrm{t}\mathrm{i}\mathrm{v}\mathrm{i}\mathrm{t}\mathrm{y}=\left(\frac{\mathrm{A}+\mathrm{B}}{2}\right)-\left(\frac{\mathrm{C}+\mathrm{D}}{2}\right)$$

### Malt determination

#### Physical properties determination

##### Malting weight loss (MWL)

Malting weight loss determination involved calculating the differential between dry matter-based 1000-kernel weights of intact finger millet grain and processed dried polished malt using Eq. (4). This gravimetric analysis quantifies the mass reduction occurring during the malting process through metabolic consumption of seed reserves.

4 $$\:\mathrm{M}\mathrm{W}\mathrm{L}\left(\mathrm{\%}\right)=\left(\frac{\mathrm{T}\mathrm{K}\mathrm{W}\mathrm{G}-\mathrm{T}\mathrm{K}\mathrm{W}\mathrm{M}}{\mathrm{T}\mathrm{K}\mathrm{W}\mathrm{G}}\right)\times\:100$$

Where:TKWG = 1000 kernel weight of grain.

TKWM = 1000 kernel weight of malt.

##### Milling

Fine-grind malt milling was carried out using a standard miller (AOAC, 2023 Method 935.30). Then, the malt flour samples were analyzed for the following parameters:

##### Wort color

The color value of the wort for each sample was determined using UV-VIS spectrophotometer (Model UV – 1800-part No. 220–92961-01, Japan) EBC (2023). A gas free and filtered wort (10 mL) was transferred into a cuvette to measure the color of the wort at 430 nm wavelength using the following Eq. (5)

Wort color (^o^EBC) = 25*A430 …………..(5); Where A_430 =_ Absorbance at 430 nanometers.

25 = multiplication factor.

**Viscosity (v): -** Wort viscosity determination utilized an Anton Paar Lovis 2000 automated falling ball viscometer following ASBC Wort-13B methodology^23^. The instrument underwent cleaning, drying, and water level calibration with jacketed temperature control at 20 °C. Degassed wort sample (40 mL) was introduced into the measuring tube with 20 mm headspace. Dual falling balls were positioned before sealing and thermal equilibration for 20–30 min. The measuring tube was secured using the locking pin mechanism. Triplicate falling time measurements ensured statistical reliability of viscosity calculations.

The viscosity was calculated as the mean values of the two balls using the following Eqs. (6)-(8):

6 $$\:{\mathrm{V}}_{\mathrm{f}}=({\mathrm{D}}_{\mathrm{b}}-{\mathrm{D}}_{\mathrm{w}})\times\:{\mathrm{T}}_{\mathrm{f}}\times\:{\mathrm{K}}_{\mathrm{f}}$$

7 $$\:{\mathrm{V}}_{\mathrm{s}}=({\mathrm{D}}_{\mathrm{b}}-{\mathrm{D}}_{\mathrm{w}})\times\:{\mathrm{T}}_{\mathrm{s}}\times\:{\mathrm{K}}_{\mathrm{s}}$$

8 $$\:{\mathrm{V}}_{\mathrm{a}}=\left(\frac{{\mathrm{V}}_{\mathrm{f}}+{\mathrm{V}}_{\mathrm{s}}}{2}\right)$$

Where: $\:{\mathrm{V}}_{\mathrm{f}}$ is the viscosity of the fastball, $\:{\mathrm{D}}_{\mathrm{b}}$ is the density of ball common for the fast and slow ball (2.232), $\:{\mathrm{D}}_{\mathrm{w}}$ is the density of the wort (common for two balls), $\:{\mathrm{T}}_{\mathrm{f}}$ is the falling time of the fastball in second,$\:\:{\mathrm{K}}_{\mathrm{f}}$ is the constant for a fastball (0.079308), $\:{\mathrm{V}}_{\mathrm{s}}$ is the viscosity of slow ball, $\:{\mathrm{T}}_{\mathrm{s}}$ is the falling time of slow ball (in second),$\:\:{\mathrm{K}}_{\mathrm{s}}$ is the constant for the slow ball (0.0098605) and $\:{\mathrm{V}}_{\mathrm{a}}$is the average viscosity. The result was reported in centipoises (cP).

#### Chemical properties

##### Moisture content determination

Moisture content analysis involved precise weighing of finger millet malt flour (4 g) using an analytical balance, followed by thermal desiccation at 105 °C for 4 h. Moisture loss calculation utilized Eq. (9) and was expressed as percentage following AOAC (2000) method 44–15 A protocols^24^.

9 $$\:\mathrm{M}\mathrm{C}\left(\mathrm{\%}\right)=\left(\frac{\mathrm{W}_{2}-\mathrm{W}_3}{\mathrm{W}_2-\mathrm{W}_1}\right)\mathrm{x}100$$

Where:

MC = moisture content of the malt.

W_1_ = weight of the container.

W_2_ = Weight of the sample and container.

W_3_ = weight of the sample and container after drying.

##### The protein content

Nitrogen content determination employed the micro-Kjeldahl method following AACC (2000) Method 46 − 11 protocols^25^. Flour samples (1 g) were weighed into Kjeldahl digestion flasks with catalyst mixture addition (1 g K_2_SO_4_ and CuSO_4_.5H_2_O per flask). Concentrated H_2_SO_4_ (15 mL, 98%) was added, and samples underwent digestion at 350 °C until achieving clear white solutions.

Following digestion completion and cooling, distilled water (50 mL) and 40% NaOH (50 mL) were sequentially added. The mixture underwent immediate distillation with the digestion tube inserted into receiver flasks containing 25 mL of 4% boric acid solution. Collected ammonia distillate was titrated against standardized 0.1 N HCl to endpoint determination.

Nitrogen percentage conversion to protein content utilized the 6.25 conversion factor. Nitrogen content calculations followed Eqs. (10) and (11) for accurate protein quantification.

10 $$\:\mathrm{P}\mathrm{r}\mathrm{o}\mathrm{t}\mathrm{e}\mathrm{i}\mathrm{n}\:\left({\%}\right)=\mathrm{N}\:{\times}\:\mathrm{F}$$

11 $$\:\mathrm{N}\mathrm{i}\mathrm{t}\mathrm{r}\mathrm{o}\mathrm{g}\mathrm{e}\mathrm{n}\left(\%\right)=\left[\frac{\left({\mathrm{V}}_{2}-{\mathrm{V}}_{1}\right)-\mathrm{B}\mathrm{*}0.1\mathrm{*}14.00}{\mathrm{M}}\right]$$

Where:

V_2_ = final volume of HCl consumed at end point of the titration in milliliter.

V_1_ = initial volume of HCL.

B = volume of 0.1 N HCL consumed for blank.

0.1 = normality of HCL.

14.00 = molecular weight of nitrogen.

M = weight of the sample.

N = percent of nitrogen.

F = conversion factor (6.25).

##### Determination of total phenolic compounds

Total phenolic content determination in finger millet grain and malt samples employed the Folin-Ciocalteu colorimetric method^26^. Malt samples (5 g) underwent homogenization with 10 mL of 60% methanolic solution containing 0.1% HCl, followed by water bath extraction at 85 °C for 2 h to eliminate ascorbic acid interference.

The settled solution was transferred to a 100 mL volumetric flask and diluted to volume with distilled water. Extract filtration utilized Whatman No. 1 filter paper under reduced pressure conditions. The colorimetric assay combined 5 mL extract with 15 mL distilled water, 5 mL Folin-Ciocalteu reagent, and 10 mL saturated sodium carbonate solution, with final volume adjustment using distilled water.

After 30-minute ambient temperature incubation, absorbance measurement occurred at 750 nm using UV-spectrophotometry (Agilent Model G6860A, Malaysia). Triplicate determinations ensured analytical precision, with quantification based on gallic acid calibration curves spanning 0–5 mg/mL concentrations. Linear regression analysis yielded excellent correlation (R² = 0.9989) with absorbance values ranging 0.0–0.5.0.5. Total phenolic content expression utilized milligrams gallic acid equivalents per 100 g (mg GAE/100 g) through Eq. (12) calculations.

12 $$C\:\left(\frac{\mathrm{m}\mathrm{g}\:\mathrm{G}\mathrm{A}\mathrm{E}}{100\mathrm{g}}\right)=\left(\frac{{C\:}_{1}V}{m}\right)$$.

Where.

C = total phenolic content in mg/g, in GAE (Gallic acid equivalent);

C_1_ = the concentration of gallic acid established from the calibration curve in mg/ml (y = 0.1395x + 0.1908; R^2^ = 0.9989); - and.

V = the volume of extract in ml; m = the weight of extract in gram.

##### Tannin content

Tannin quantification in finger millet grain and malt utilized the modified Vanillin-HCl colorimetric assay developed by Schofield et al., 2001^27^. Finger millet flour samples (0.15 g) were transferred to 15 mL centrifuge tubes and extracted with 4 mL of 1% methanolic HCl solution.

Extraction proceeded at 30 °C for 30 min in a water bath, followed by centrifugation at 4000 rpm for 10 min. The supernatant (1 mL) was aliquoted into duplicate tubes for sample and control analyses. Working vanillin reagent (5 mL) was added to sample tubes, while control tubes received 5 mL of 4% methanolic HCl. Both preparations underwent thermal incubation at 30 °C for 20 min.

Catechin calibration standards were prepared up to 0.6 mg/mL with serial dilutions. Spectrophotometric measurements of samples, controls, and standards were performed using UV-VIS spectrophotometry (Agilent Model G6860A, Malaysia) at 500 nm for precise tannin determination. Then the tannin content was calculated using Eq. (13).

13 $$Tannin \:concentration (mg\: catechin \:equivalent/g) =\:\left(\frac{V*A/m}{Ws}\right)$$

Where:

V = Volume of extract in mL (8 mL);

A = Absorbance at 500 nm (absorbance of sample minus absorbance of blank);

m = slope of the standard curve from catechin equivalent; and.

Ws = Weight of sample (0.15 g).

##### Calcium

Calcium content was determined by the classic method of determining calcium and other suitable cations titration with a standardized solution of ethylene diamine tetra acetic acid (EDTA). Wort sample (10 ml), 100 ml of distilled water, and 3 ml of sodium hydroxide (8 N) were mixed and four drops of red regent were added. Then it titrated with EDTA to end point, and color was changed from pink to gray blue color. The result was calculated as consumed volume times 40.08. The result is expressed in mg/100 g dry weight basis for malt samples according to the Heineken Brewery procedure.

##### Diastatic power

Diastatic power determination in finger millet malt employed EBC method 4.12 (2023) methodology as described by A. Tura et al. (2024)^28^. Finely ground malt (20 g) was weighed into stainless steel containers and extracted with cold distilled water (450 mL) at 40 °C under continuous stirring for one hour. The extraction was cooled and adjusted to 520 g total weight, followed by filtration with disposal of the initial 200 mL filtrate and collection of the subsequent 50 mL for analysis.

Four graduated flasks (200 mL each) were prepared: flasks 1–2 for primary analysis and flasks 3–4 for blank controls. Each flask received 100 mL starch solution, with acetate buffer (5 mL) added exclusively to flasks 1–2. All flasks underwent thermal equilibration at 20 °C for 20 min. Malt extract (5 mL) was sequentially pipetted into flasks 1 and 2 with precise one-minute intervals, followed by vigorous mixing and 30-minute incubation from initial extract addition.

Enzymatic inactivation occurred through sodium hydroxide addition: 4 mL to flasks 1–2, and 2.35 mL to flasks 3–4, with subsequent 5 mL extract addition to blank flasks. All solutions were diluted to 200 mL and confirmed alkaline using thymolphthalein indicator.

Iodometric titration involved transferring 50 mL aliquots from each flask to corresponding 150 mL conical flasks. Iodine solution (25 mL) and sodium hydroxide (3 mL) were added, followed by 15-minute incubation under loose glass stoppers to prevent iodine volatilization. Acidification with sulfuric acid (4.5 mL) preceded titration of unreacted iodine with sodium thiosulfate to colorless endpoint.

Calculations expressed results as maltose equivalents produced by 100 g malt under standardized conditions. The corrected iodine consumption (sample minus blank) was multiplied by 34.2 for Windisch-Kolbach unit expression. Conversion to Lintner degrees utilized the regression equation: Lintner degrees = 0.3 × Windisch-Kolbach units + 4.

##### Hot water extract (HWE)

The extract was determined after the congress mash was done in a mash bath. Milled finger millet malt (50 g) was taken. Distilled water (200 ml) was transferred to a 500 ml beaker. The mash bath was powered on, and the distilled water (200 ml) was warm (45^*°*^C). The prepared 50 g of each sample flour was transferred to beakers in the warm mash bath and stirred and mixed with water. The mixture of the sample flour and water was continuously stirred for 30 min in a mash bath set at 45^*°*^C. Then the temperature of the mash was raised by 1^*°*^C per minute for 30 min up to 70^*°*^C. The mash was kept at 70^*°*^C for a further 60 min in the bath water under stirring. Saccharification monitoring involved iodine testing using 0.02 N iodine solution after 10 min. Upon mashing completion, samples underwent ambient temperature cooling followed by distilled water adjustment to achieve 450 g total mash vessel weight.

Wort density determination utilized portable densitometry (DMA35 basic, Austria) on clarified wort samples, with results expressed in degrees Plato (°P). Extract yield calculations were converted to dry weight basis percentages for standardized reporting. The extract content of malt was calculated using Eqs. (14) and (15)

14 $$\:{\mathrm{E}}_{1}=\frac{\mathrm{P}\mathrm{*}\left(\mathrm{M}+800\right)}{100-\mathrm{P}}$$

15 $$\:{E}_{2}=\frac{E1\mathrm{*}100}{100-M}$$

Where:

E_1_ **=** the extract content of malt sample taken, in %(m/m).

E_2_ **=** the extract content of dry malt, in%(m/m).

P **=** the extracted content in the wort, in g of extract per 100 g of wort (=% Plato).

M **=** the moisture content of the malt, in % (m/m).

800 = the amount of distilled water added into the mash to 100 g of malt.

##### Free amino nitrogen (FAN)

Free amino nitrogen (FAN) quantification employed the modified Ninhydrin colorimetric method following ASBC Wort-12 protocols (2008). Wort samples (1.0 mL) underwent dilution to 100 mL with distilled water in volumetric flasks. Duplicate aliquots (2 mL) were transferred to test tubes with ninhydrin solution addition (1 mL). Thermal treatment involved 16-minute boiling water bath incubation followed by 20-minute cooling in cold water bath. Potassium iodate dilution solution (5 mL) was added to maintain ninhydrin oxidation state. Spectrophotometric analysis occurred at 570 nm wavelength. Analytical calculations utilized average duplicate absorbance readings with blank subtraction from both samples and glycine standards. FAN values (mg/L) were calculated using Eq. (16) for precise nitrogen quantification.

16 $$\:\mathrm{F}\mathrm{A}\mathrm{N}\left(\frac{\mathrm{m}\mathrm{g}}{\mathrm{L}}\right)=\left[\frac{2\mathrm{d}\left({\mathrm{A}}_{t}-{\mathrm{A}}_{b}-{\mathrm{A}}_{c}\right)}{{\mathrm{A}}_{s}-{A}_{b}}\right]$$

Where: 2 = concentration of glycine standard solution (in mg per L).

d = dilution factor (dilution was 1 ml to 100 ml so d = 100).

A_t_ = Average absorbance of diluted wort sample.

A_b =_ Average absorbance of blanks.

A_c =_ Average absorbance for correction of dark colored samples.

A_s =_ Average absorbance of glycine standard

$$\:\mathrm{F}\mathrm{r}\mathrm{e}\mathrm{e}\:\mathrm{a}\mathrm{m}\mathrm{i}\mathrm{n}\mathrm{o}\:\mathrm{n}\mathrm{i}\mathrm{t}\mathrm{r}\mathrm{o}\mathrm{g}\mathrm{e}\mathrm{n}\:\left(\mathrm{F}\mathrm{A}\mathrm{N}\right),\raisebox{1ex}{$\mathrm{m}\mathrm{g}$}\!\left/\:\!\raisebox{-1ex}{$\mathrm{L}$}\right.=solution\:net\times\:2\times\:\mathrm{d}\mathrm{i}\mathrm{l}\mathrm{u}\mathrm{t}\mathrm{i}\mathrm{o}\mathrm{n}$$.

##### Kolbach index (ratio S/T)

Kolbach index was calculated according to ASBC (2008) by using Eq. (17).

Soluble nitrogen content in 1 l wort was calculated using Eq. (17)

17 $$\:{N}_{v}=\left(\frac{T\mathrm{*}14\mathrm{*}100}{V}\right)$$

Where, N_V_= soluble nitrogen content in 1 l wort (mg/L).

T = Acid titration value with blank subtracted corrected to exactly one tenth normality.

14 = Atomic weight of nitrogen

V = sample volume.

100 = correction factor to obtain the result per 1 l (ml/L) soluble nitrogen content as percentage of dry malt was obtained from Eq. (18)

18 $$\:{S}_{v}=\left(\frac{{N}_{v}\mathrm{*}{E}^{{\prime\:}}}{100\mathrm{*}{E}_{w}}\right)$$

Where, Ns = soluble nitrogen content as a percentage of dry malt (%).

Nv = soluble nitrogen content (mg/L).

E’ = Extract of dry malt (%).

E_w_ = Grams of extract in 100 ml wort (g/100 ml).

100 = correction factor to obtain the result as a percentage

Soluble nitrogen content as a percentage of the total nitrogen (%) (Kolbach index) was calculated using Eq. (19)

19 $$\:{N}_{k}=\left(\frac{Ns\mathrm{*}100}{N}\right)$$

Where, N_K_ = soluble nitrogen content as a percentage of total nitrogen (%) kolbach index.

Ns = soluble nitrogen content as a percentage of dry malt %.

N = Total nitrogen content as a percentage of dry malt %.

##### Wort pH

The pH of the wort was measured according to the method described by^26^ using a digital electronic pH meter, after calibrating with standard buffer solutions of pH 4, 7, and 10.

## Results and discussion

### Quality parameters of raw finger millet grain

#### Germination energy (GE)

**Fig. 1:**
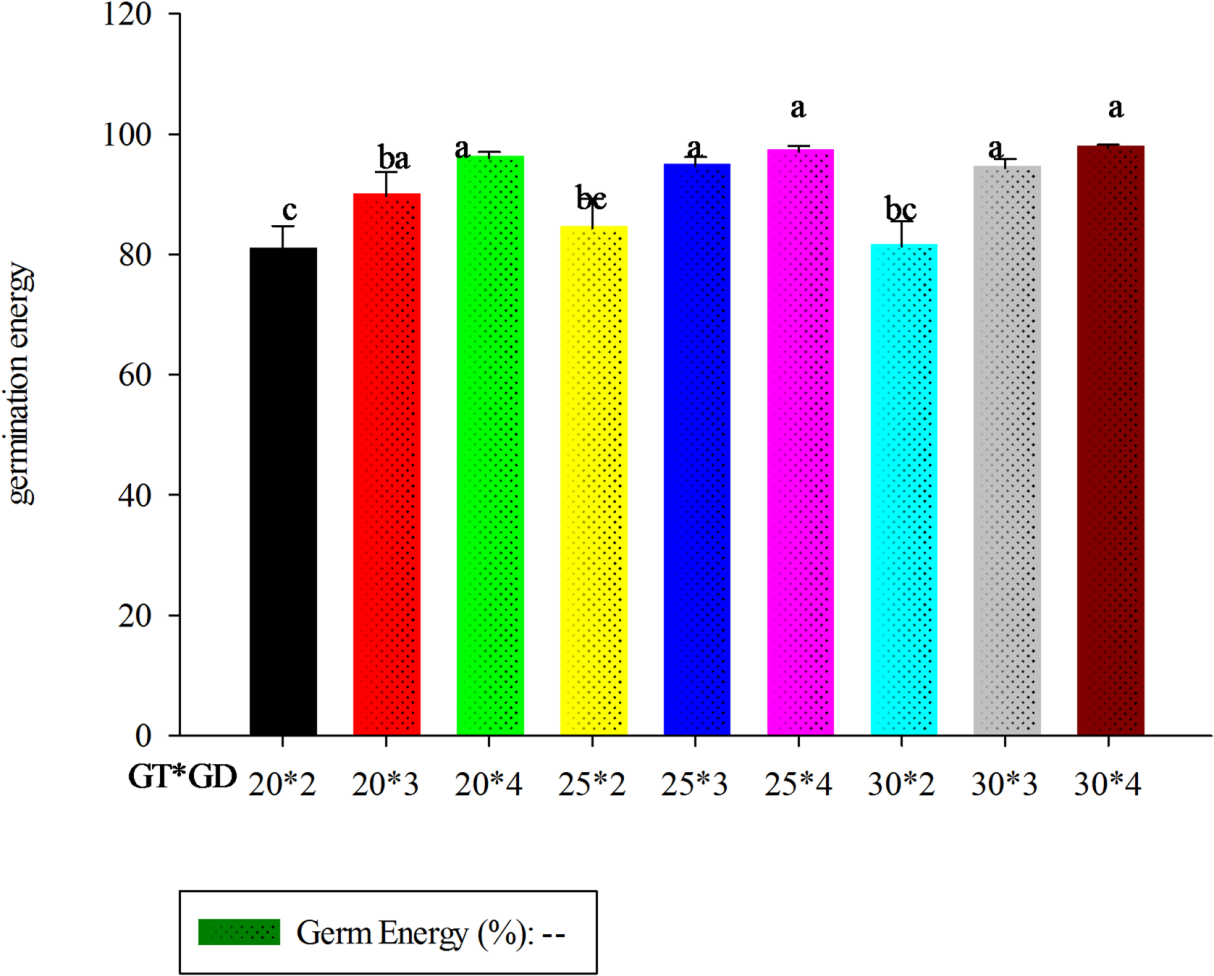
Interaction effect of germination temperature and germination time on GE.

Germination energy is the total number of grains that germinate over a given time of incubation under specified condition^30^. As indicated in Fig. 1, the highest germination energy value (97.66%) was obtained in a sample germinated at 30^*°*^C for 4 days whereas the smallest value (80.66%) was recorded at 20^*°*^C for 2 days. Germination energy increased as germination day increased from day one to 4th day. There was no significant difference among the germination temperatures used for the study. The hectoliter weight values met established quality standards (Appendix-A)^31^. The germination energy for finger millet should be at least 90% at 72 h germination times to be accepted for malting^32^. The result obtained in the current study fulfills the recommended value.

#### Water sensitivity (WS)

**Fig. 2:**
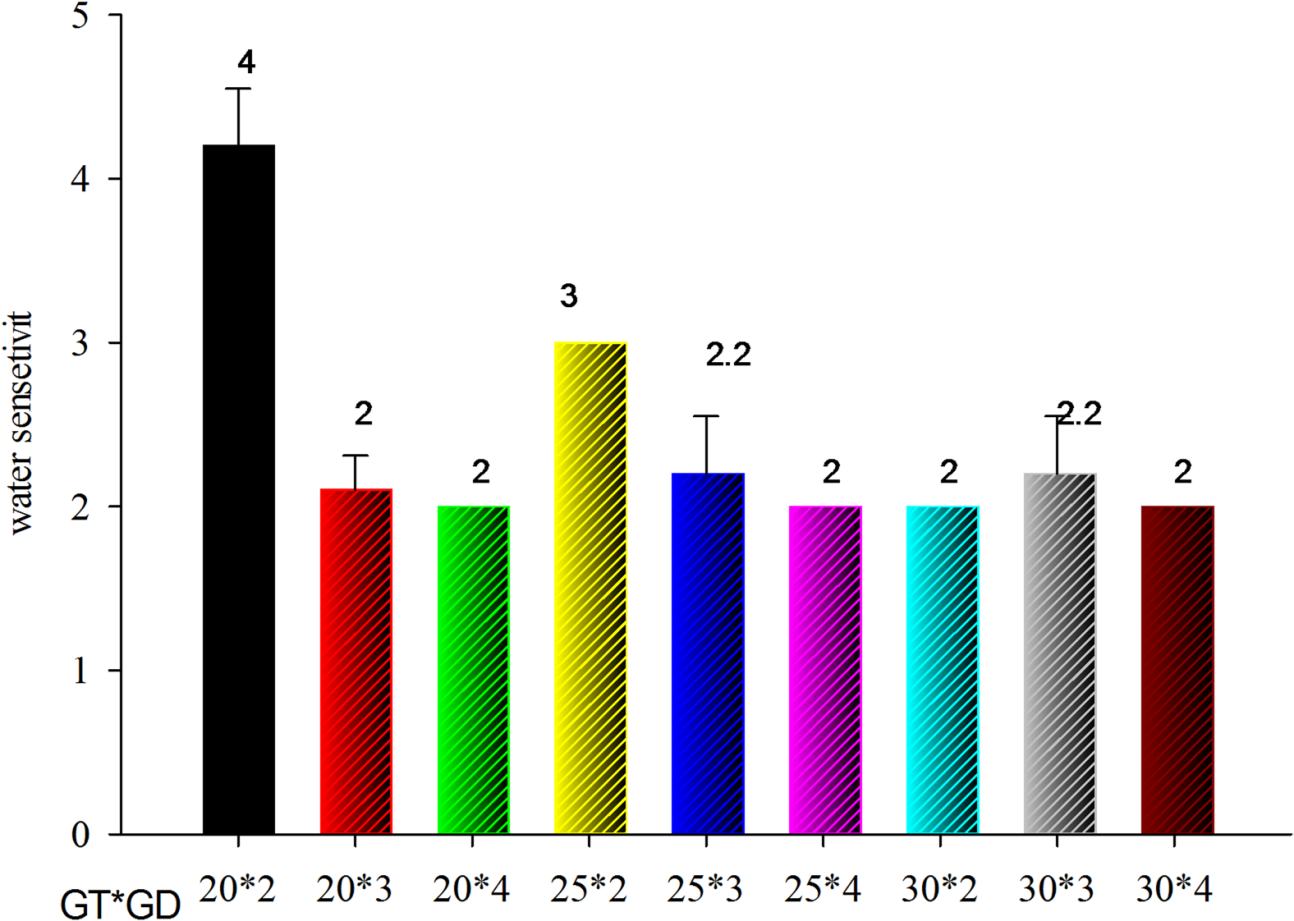
Interaction effect of germination temperature (GT) and germination day (GD) on WS.

Water sensitivity showed a decrement from 2.8 to 2.0 with an increment in germination temperature from 20^*°*^C to 30^*°*^C. WS is also affected by germination day, which decreased from 3.0 to 2.0 as germination day increased from day one to day three, but the difference between two days and three days is not statistically significant. The highest WS value (4) was obtained in a sample germinated at 20^*°*^C for 1 day, whereas the lowest value (2.0) was shown at 30^*°*^C for 4 days shown in Fig. 2.

### Finger millet malt quality

#### Hectoliter weight of malt

The three-way interaction showed significant effects (F = 12.45, df = 18, *p* < 0.001) on hectoliter weight. The highest (73.75 kg/hL) hectoliter weight value of finger millet malt was obtained in a sample germinated at 20^*°*^C for two days and cured at 80^*°*^C whereas the lowest value (66.37 kg/hL) was recorded at 25^*°*^C for 3 days and cured at a temperature of 90^*°*^C. All the malt samples fulfilled the EBC standard requirement set for hectoliter weight of barley malt. According to the EBC standard, a hectoliter weight of 65–75 kg/hL is required. In Ethiopia, Dashen Brewery Share Company requires a hectoliter weight of 75 kg/hL for grade 1, 70 kg/hL for grade 2, and 68 kg/hL for grade 3. The hectoliter weight values met established quality standards (Appendix-A)^31^. The finding of the current study revealed that the hectoliter weight ranged from 66.37 to 73.74 kg/hL. Thus, the result indicates suitability of the treatment combinations for malt production.

**Table 1:** Effect of GT, Gt and CT on some physical quality parameters of malt.

Factors			Parameters		
GT	Gt	CT	Hectoliter weight	1000 kernel weight	Malting weight loss
20	2	70	73.41 ± 0.005^b^	2.49 ± 0.0009^a^	20.28 ± 0.03^m^
		80	73.740 ± 0.0003^a^	2.42 ± 0.0008^e^	22.50 ± 0.02^h^
		90	73.40 ± 0.0^b^	2.40 ± 0.0001^g^	22.96 ± 0.005^ed^
	3	70	72.38 ± 0.005^edf^	2.47 ± 0.001^b^	20.73 ± 0.04^l^
		80	70.61 ± 0.001^i^	2.41 ± 0.0005^f^	22.83 ± 0.01^g^
		90	70.82 ± 0.0003^ih^	2.40 ± 0.00001^g^	22.97 ± 0.00^ed^
	4	70	70.79 ± 0.0.0003^ih^	2.46 ± 0.002^c^	21.11 ± 0.06^k^
		80	68.43 ± 0.23^m^	2.41 ± 0.0002^f^	22.85 ± 0.01^fg^
		90	71.02 ± 0.0001^h^	2.40 ± 0.0^g^	22.97 ± ^e^0.005^d^
25	2	70	72.61 ± 0.019^d^	2.46 ± 0.0001^c^	21.15 ± 0.005^k^
		80	72.17 ± 0.015^gf^	2.41 ± 0.0001^f^	22.90 ± 0.005^efg^
		90	72.98 ± 0.000^c^	2.40 ± 0.00003^g^	22.97 ± 0.005^ed^
	3	70	70.97 ± 0.220^h^	2.44 ± 0.001^d^	21.72 ± 0.05^j^
		80	67.71 ± 0.103^n^	2.41 ± 0.0001^f^	22.90 ± 0.005^ef^
		90	66.37 ± 0.0^p^	2.40 ± 0.00002^g^	22.99 ± 0.00^d^
	4	70	69.92 ± 0.318^j^	2.44 ± 0.0001^d^	21.73 ± 0.005^j^
		80	67.11 ± 0.002^o^	2.40 ± 0.0001^g^	22.95 ± 0.00^ed^
		90	67.16 ± 0.0005^o^	2.39 ± 0.0001^g^	23.22 ± 0.03^c^
30	2	70	71.87 ± 0.157^g^	2.44 ± 0.0002^d^	21.76 ± 0.01^j^
		80	72.51 ± 0.0003^ed^	2.400.0001 ± ^g^	22.95 ± 0.005^ed^
		90	71.87 ± 0.007^g^	2.39 ± 0.0001^h^	23.33 ± 0.005^b^
	3	70	70.91 ± 0.0^ih^	2.44 ± 0.0002^d^	21.78 ± 0.005^j^
		80	70.00 ± 0.0^j^	2.40 ± 0.00005^g^	22.96 ± 0.00^ed^
		90	68.99 ± 0.003^l^	2.39 ± 0.0001^h^	23.34 ± 0.00^b^
	4	70	68.46 ± 0.04^m^	2.44 ± 0.0002^d^	21.92 ± 0.01^i^
		80	69.59 ± 0.03^k^	2.40 ± 0.0001^g^	22.96 ± 0.00^ed^
		90	67.88 ± 0.018^n^	2.35 ± 0.0008^i^	24.51 ± 0.02^a^
Pv			< 0.0001	< 0.0001	<0.0001
CV			0.13	0.06	0.10

Mean values not followed with the same letter in a column are significantly different at *P* < 0.05.

Abbreviation: GT = germination temperature; Gt = germination time; CT = Curing temperature.

#### Thousands kernel weight (TKW)

The TKW was significantly (*p* < 0.05) affected by germination temperature, germination time, and curing temperature. It decreased as the germination temperature rose. The TKW of finger millet malt also decreased in the same fashion when both germination day and curing temperature increased. The maximum thousand kernel weight value (2.49 gm) was obtained in malt sample germinated at 20^*°*^C for two days and cured at 70^*°*^C whereas the lowest value (2.35gm) was recorded at 30^*°*^C for 4 days and cured at 90^*°*^C as stated in Table 1. At higher curing temperature lower thousands kernel weight was recorded this is because of higher removal of moisture content from the malt at higher curing temperature. Malt germinated in longer germination time absorbs more water than shorter germination time and then release more water during curing and cause decrement of thousand kernel weight. As malt grain is exposed to higher germination temperature and longer germination time the porosity (the more modified the grain the higher the porosity) of the grain increases and leads to decline of thousand kernel weight.

#### Malting weight loss (MWL)

The MWL of finger millet malt was significantly affected (*p* < 0.05) by germination time and curing temperature. MWL increased with an increment of germination time. MWL increased as the curing temperature increased. This might be due to the loss of moisture content. In this work, the smallest value (20.28%) of malting weight loss was obtained for malt samples germinated at 20^*°*^C for two days and cured at a temperature of 70^*°*^C whereas the highest value (24.51%) was recorded for malt samples germinated at 30^*°*^C for 4 days and cured at a temperature of 90^*°*^C as indicated in Table 1. The MWL can be manifested by the loss of soluble substances in steeping, respiration during germination and removal of rootlets from the malt^33^. The malting weight loss in the range of 6.5–10.5%^34^. In the current study, the MWL ranged from 20.28 to 24.51%, which is much higher than the malting weight loss. It is due to several factors specific to finger millet and our experimental conditions: the smaller grain size resulted in higher surface area-to-volume ratios increasing moisture loss, the extended germination periods promoted greater metabolic activity, and the Mecha variety may have inherently different reserve utilization patterns compared to barley or other cereals studied in previous research. The MWL increased with germination time and increasing moisture content^34^.

#### Malt moisture content (MMC)

The MMC was significantly affected (*p* < 0.05) by the interaction effect of germination temperature, germination time and curing temperature. The moisture content of the malt varied from 2.07 to 4.48% in all the treatment combinations. The highest value (4.48%) was noticed in malt sample germinated at a temperature of 20^*°*^C for two days and cured at 70^*°*^C, whereas the smallest MMC (2.07%) was recorded for malt sample germinated at a temperature of 30^*°*^C for 4 days and cured at 90^*°*^C as indicated in Table 2. Lowest MCC was obtained at higher (90^*°*^c) because higher temperature leads to removal of more water from malt during curing.

Malt moisture content typically ranges from 1.5 to 6% on fresh weight basis^35^. Industrial quality standards establish maximum moisture specifications: Asella Malt Factory requires ≤ 5.8%, while EBC standards accept 3–5.8.8% moisture content for acceptable malt quality.

The maximum finger millet malt moisture content (4.48%) observed in this investigation aligns with both EBC and Asella Malt Factory specifications. These values correspond closely with previous findings by Owheruo et al. (2019)^36^, who documented finger millet malt moisture content ranging from 2.16 to 4.50%, confirming the consistency of moisture levels across different finger millet malt preparations.

**Table 2:** Effect of GT, Gt and CT on some chemical malt quality traits.

Factors			Parameters		
GT	Gt	CT	Moisture Content (%)	Total Protein (%)	Kolbach Index (%)
20	2	70	4.48 ± 0.13^a^	7.94 ± 0.073^dc^	14.33 ± 0.006^ih^
		80	2.18 ± 0.05^cbd^	7.82 ± 0.071^edc^	15.33 ± 0.014^gfh^
		90	2.19 ± 0.13^cbd^	8.11 ± 0.108^b^	16.05 ±0.002^gfehd^
	3	70	2.69 ± 0.14^cb^	7.66 ± 0.115^ejdf^	14.66 ± 0.0020^i^
		80	2.14 ± 0.06^cd^	7.61 ± 0.053^hegdf^	15.30 ± 0.0060^gih^
		90	2.29 ± 0.13^cbd^	7.63 ± 0.177^hegdf^	16.40 ± 0.003^gfehd^
	4	70	2.66 ± 0.22^cbd^	7.56 ± 0.108^hegf^	17.26 ± 0.002^fecd^
		80	2.10 ± 0.01^cd^	7.78 ± 0.0241^edc^	15.70 ± 0.0004^gih^
		90	2.21 ± 0.01^cbd^	8.21 ± 0.0^ba^	15.30 ± 0.0002^gih^
25	2	70	2.76 ± 0.31^b^	7.49 ± 0.151^hegf^	15.20 ± 0.004^gfeh^
		80	2.19 ± 0.12^cbd^	6.66 ± 0.224^jk^	16.20 ± 0.0005^gfecd^
		90	2.20 ± 0.46^cbd^	7.36 ± 0.070^hg^	16.04 ± 0.001^gfehd^
	3	70	2.34 ± 0.23^cbd^	8.32 ± 0.109^a^	15.07 ± 0.005^i^
		80	2.34 ± 0.03^cbd^	6.69 ± 0.108^jk^	18.04 ± 0.002^bc^
		90	2.35 ± 0.24^cbd^	7.38 ± 0.045^hg^	17.66 ± 0.001^bcd^
	4	70	2.29 ± 0.21^cbd^	7.81 ± 0.098^edc^	16.00 ± 0.002^gfecd^
		80	2.45 ± 0.29^cbd^	8.28 ± 0.152^ba^	14.34 ± 0.002^ih^
		90	2.36 ± 0.15^cbd^	7.78 ± 0.024^edc^	17.00 ± 0.003^fecd^
30	2	70	2.33 ± 0.32^cbd^	6.52 ± 0.019^k^	20.00 ± 0.0005^a^
		80	2.27 ± 0.17^cbd^	6.73 ± 0.058^jk^	15.40 ± 0.007^gfeh^
		90	2.08 ± 0.06^d^	7.30 ± 0.129^hi^	17.20 ± 0.003^bcd^
	3	70	2.25 ± 0.15^cbd^	6.45 ± 0.109^k^	20.05 ± 0.003^a^
		80	2.09 ± 0.04^d^	6.47 ± 0.068^k^	17.30 ± 0.007^becd^
		90	2.08 ± 0.05^d^	6.97 ± 0.042^ji^	19.08 ± 0.001^ba^
	4	70	2.21 ± 0.17^cbd^	7.73 ± 0.236^edf^	16.00 ± 0.005^gfecd^
		80	2.19 ± 0.13^cbd^	7.78 ± 0.024^edc^	19.00 ± 0.0005^ba^
		90	2.07 ± 0.05^d^	7.41 ± 0.044^hgf^	17.66 ± 0.001^bcd^
Pv			< 0.0001	< 0.0001	< 0.0001
CV			0.13	1.44	3.28

Mean values not followed with the same letter in a column are significantly different at *P* < 0.05.

Abbreviation: GT = germination temperature; Gt = germination time; CT = curing temperature.

#### Total protein content (TPC)

The analysis showed there was a significant difference (*p* < 0.05) among the combinations for crude protein content. The mean maximum value (8.32%) of malt protein content was recorded in a malt sample germinated at 25^*°*^C for three days and cured at 70^*°*^C followed by the malt value (8.11%) germinated at 20^*°*^C for three days and cured at 90^*°*^C. The minimum total protein content (6.45%) was obtained at a malt sample germinated at 30^*°*^C for three days and cured at 70^*°*^C (Table 2). The maximum protein value (8,32%) in this finding is close to the minimum protein value (9%) set by EBC standard protein content for barley.

Ethiopian finger millet variety analysis by Admassu et al. (2009)^37^ documented protein content ranging from 6.26 to 10.50 mg/100 g across nine cultivars. Elevated protein content in malt grain presents processing challenges due to proportionally reduced carbohydrate content, resulting in decreased malt extract yields^38^.

Excessive protein levels extend grain modification duration during malting, promoting increased rootlet development and elevated respiratory and metabolic losses^39^. High-protein grains typically contain greater concentrations of soluble protein and albuminoid materials compared to low-protein counterparts. These soluble proteins transfer into wort extracts, potentially causing haze formation and compromising beer stability^38^.

Conversely, insufficient protein content limits essential nutrients required for optimal yeast metabolism during fermentation processes^40^. Therefore, balanced protein levels are crucial for maintaining both extract quality and fermentation efficiency in malting applications.

#### Kolbach index (KI)

Kolbach index is a measure of the extent of protein modification in beer. It comprises the assessment of total soluble protein in wort as a percentage of the total protein measureable in the malt^41^. Kolbach index was significantly (*p* < 0.05) affected by germination temperature, germination day and curing temperature. The KI increased as germination temperature increased. This is due to the decrement of soluble protein content of finger millet malt in this study. As germination day increased from 2 days to 3 days kolbach index was raised but, reduced back as germination day gone to 4 days without significant difference with malt sample germinated for 2 days. In case of three-way interaction, the kolbach index ranged from 14.33 to 20%. Malt germinated at 20^°^C for 2 days and cured at 70^°^C was the lowest without statistical difference with the malt sample germinated at 25^°^C for 4 days and cured at 80^°^C. Malt sample germinated at 30^°^C for 2 days and cured at 70^°^C was the highest. Because the smallest total protein content was obtained at malt sample germinated at 30^°^C and cured at 70^°^C. This influenced the ki which is obtained by the ratio of soluble protein content to total protein content.

According to EBC standards, the KI ranges of < 35%, 35–41% and 41–43% were under modified, well modified and very highly modified, respectively. In this study, the results obtained were below under modified range. It therefore had a lower proportion of nitrogenous materials in its extract, and this probably accounted relatively for higher fermentability^42^. Recent research by^40^ demonstrated that the KI value in the range of 41.37–45.93%. This result also showed that the interaction value of germination temperature, germination time and curing temperature was significant (*P* < 0.05) for KI, which is contradictory with the current finding. Another study carried out by^45^ showed the KI in the range of 34.49–44.72% which is significantly higher than the values of the current study.

#### Tannin content

Tannin is a polyphenol that binds to protein and influence the stability of beer^45^. The tannin content of un-germinated finger millet variety used for this study was 513.49 mg/100 g. The tannin content of the malt of this finger millet variety was obtained in the range of 337.15 to 481.62 mg/100 g (Table 3). According to^17^ report, the tannin content of un-germinated finger millet was 410 mg/100 g, 516 mg/100 g, 476 mg/100 g and 513 mg/100 g for Aksum, Meba, Tadesse and Tessema finger millet varieties, respectively. The malting conditions reduce the total tannin content. In this study, the tannin content of finger millet malts ranged from 337.15 to 481.62 mg/100 g. The highest tannin value (481.62 mg/100 g) was scored for malt sample germinated at 30^*°*^C for three days and cured at 90^*°*^C while the lowest value (337.15 mg/100 g) was observed in a malt sample germinated at 25^*°*^C for 4 days and cured at 80^*°*^C. There was reduction in tannin content after germination^46^. This reduction was a result of leaching tannins into the water; the formation of hydrophobic association of tannins with seed proteins and enzymes; and binding of polyphenols with other organic substances such as carbohydrates or protein. Apart from that, during the period of soaking prior to germination, the enzyme may be activated and result in degradation and loss of tannin^47^.

**Table 3:** Effect of GT, Gt and CT on the tannin, total phenol, and calcium content.

Factors			Parameters		
GT	Gt	CT	Tannin content	Total phenol	Calcium content
20	2	70	413.92 ± 2.04^bcdefgh^	1.48 ± 0.002^hi^	180.96 ± 0.60^h^
		80	421.01 ± 0.27^bcdefgh^	1.58 ± 0.002^c^	212.62 ± 0.20^c^
		90	431.70 ± 0.16^bcde^	1.39 ± 0.001^m^	216.83 ± 0.40^ba^
	3	70	415.26 ± 5.80^bcdefgh^	1.35 ± 0.002^o^	183.97 ± 0.40^h^
		80	424.51 ± 0.54^bcdefgh^	1.55 ± 0.002^e^	214.42 ± 2.00^bc^
		90	456.39 ± 0.41^ab^	1.45 ± 0.001^j^	217.63 ± 0.00^a^
	4	70	381.15 ± 0.71^ghi^	1.25 ± 0.002^q^	187.77 ± 0.60^g^
		80	400.63 ± 5.29^cdefgh^	1.35 ± 0.001^o^	217.03 ± 0.20^ba^
		90	407.09 ± 0.16^cdefgh^	1.34 ± 0.003^p^	217.43 ± 0.60^ba^
25	2	70	398.20 ± 0.16^cdefgh^	1.35 ± 0.002^o^	190.18 ± 0.20^ba^
		80	428.01 ± 0.27^bcdef^	1.62 ± 0.001^b^	217.43 ± 0.20^ba^
		90	434.75 ± 0.27^bcd^	1.63 ± 0.001^a^	217.43 ± 0.20^ba^
	3	70	427.48 ± 0.47^bcdef^	1.52 ± 0.002^0^	191.98 ± 0.40^f^
		80	380.43 ± 0.82^hi^	1.38 ± 0.003^n^	217.23 ± 0.40^ba^
		90	453.51 ± 0.95^ab^	1.58 ± 0.001^c^	217.03 ± 0.20^ba^
	4	70	426.94 ± 67.7^bcdefgh^	1.33 ± 0.001^p^	195.79 ± 0.60^e^
		80	337.15 ± 2.04^i^	1.39 ± 0.002^m^	217.23 ± 0.40^ba^
		90	443.82 ± 0.62^abc^	1.48 ± 0.004^i^	217.23 ± 0.40^ba^
30	2	70	428.28 ± 0.71^bcdef^	1.49 ± 0.001^h^	198.60 ± 0.20^e^
		80	441.93 ± 0.82^abc^	1.55 ± 0.002^d^	217.23 ± 0.00^ba^
		90	386.17 ± 32.2^efgh^	1.41 ± 0.002^l^	217.23 ± 0.00^ba^
	3	70	391.38 ± 0.81^defgh^	1.63 ± 0.003^a^	204.41 ± 4.01^d^
		80	442.56 ± 0.27^abc^	1.58 ± 0.004^c^	217.43 ± 0.20^bc^
		90	481.62 ± 3.00^a^	1.53 ± 0.001^f^	218.03 ± 0.40^a^
	4	70	402.16 ± 0.54^cdefgh^	1.58 ± 0.004^c^	206.41 ± 2.00^d^
		80	435.11 ± 0.41^bcd^	1.50 ± 0.001^g^	217.23 ± 0.40^ba^
		90	382.31 ± 3.15^fghi^	1.53 ± 0.002^f^	218.24 ± 0.20^a^
Pv			< 0.00	< 0.0001	< 0.00
CV			5.98	8.1234	5.98

Mean values not followed with the same letter in a column are significantly different at *P* < 0.05.

Abbreviation: GT = germination temperature; Gt = germination time; CT = curing temperature.

#### Total phenol content

Phenolic compounds undergo quantitative and qualitative transformations during germination processes^48^. Simple phenolic compounds constitute essential beer components, released from grain and hops during mashing and brewing operations^49^. Finger millet phenolic content exhibits strong correlation with grain color and geographical cultivation origin. White finger millet varieties demonstrate lower phenolic concentrations, while brown cultivars possess significantly elevated phenolic compound levels^49^.

Total phenolic content analysis revealed variation from 1.25 to 1.63 mg GAE/100 g across sample matrices. Minimum values occurred in samples germinated at 20 °C for four days with 70 °C curing, while maximum concentrations appeared in samples germinated at 30 °C for three days with 70 °C curing (Table 3). Kilning temperatures below 80 °C typically increase water-soluble phenolic compounds through Maillard-enzymatic phenol release and enhanced grain friability facilitating extraction^50^.

Polyphenolic composition serves as a critical quality indicator for beer processing and marketing applications^50^. However, phenolic type and concentration influence organoleptic properties including taste, aroma, color, colloidal stability, and foam characteristics, potentially reducing beer shelf-life. Generally, elevated total polyphenol content correlates with enhanced antioxidant activity in beverages^51^. While prenylated flavonoids demonstrate remarkable stability lasting 10 years in room-temperature beer storage, monophenols and flavonoids exhibit temperature- and time-dependent degradation patterns^50^.

#### Calcium content

Calcium serves as the principal ionic determinant of beer quality through multiple biochemical mechanisms. This essential mineral overcomes malt phosphate buffering capacity, reducing mash pH to optimal ranges while promoting clarity, flavor development, and finished beer stability^52^. Calcium’s brewing functionality extends beyond pH modification, particularly crucial for lighter beer styles where mash pH requires reduction from naturally elevated levels^53^. Additionally, calcium enhances beer quality through phosphate precipitation promotion from malt matrices, contributing significantly to finished product stability and shelf-life extension^54^.

In the current study, the calcium content of the malt samples ranged from 180.96 mg/100 g to 218.24 mg/100 g dry weight basis, with the malt germinated at 20^°^C for 3 days and cured at 70^°^C depicting the lesser and the malt germinated at 30^°^C for 4 days and cured at 90^°^C was the highest (Table 3). Because as germination temperature germination time and curing temperature raised contents like total phenol content and tannin content decreased and calcium content exposed and expressed more. But there was no statistical difference with the malt samples germinated at 30^°^C for 3 days and at 20^°^C for 3 days both cured at 90^°^C. The finger millet malt calcium content of the current finding agreed with most of the previous studies which demonstrated that calcium content between 189.93 mg/100 g to 272.36 mg/100g^55^.

### Quality parameters of finger millet wort

The quality of wort is important for producing quality beer^56^. The finger millet malt wort was analyzed for quality parameters such as wort color, wort viscosity, diastatic power, fine grind hot water extract, free amino nitrogen, and pH.

#### Wort color

**Fig. 3:**
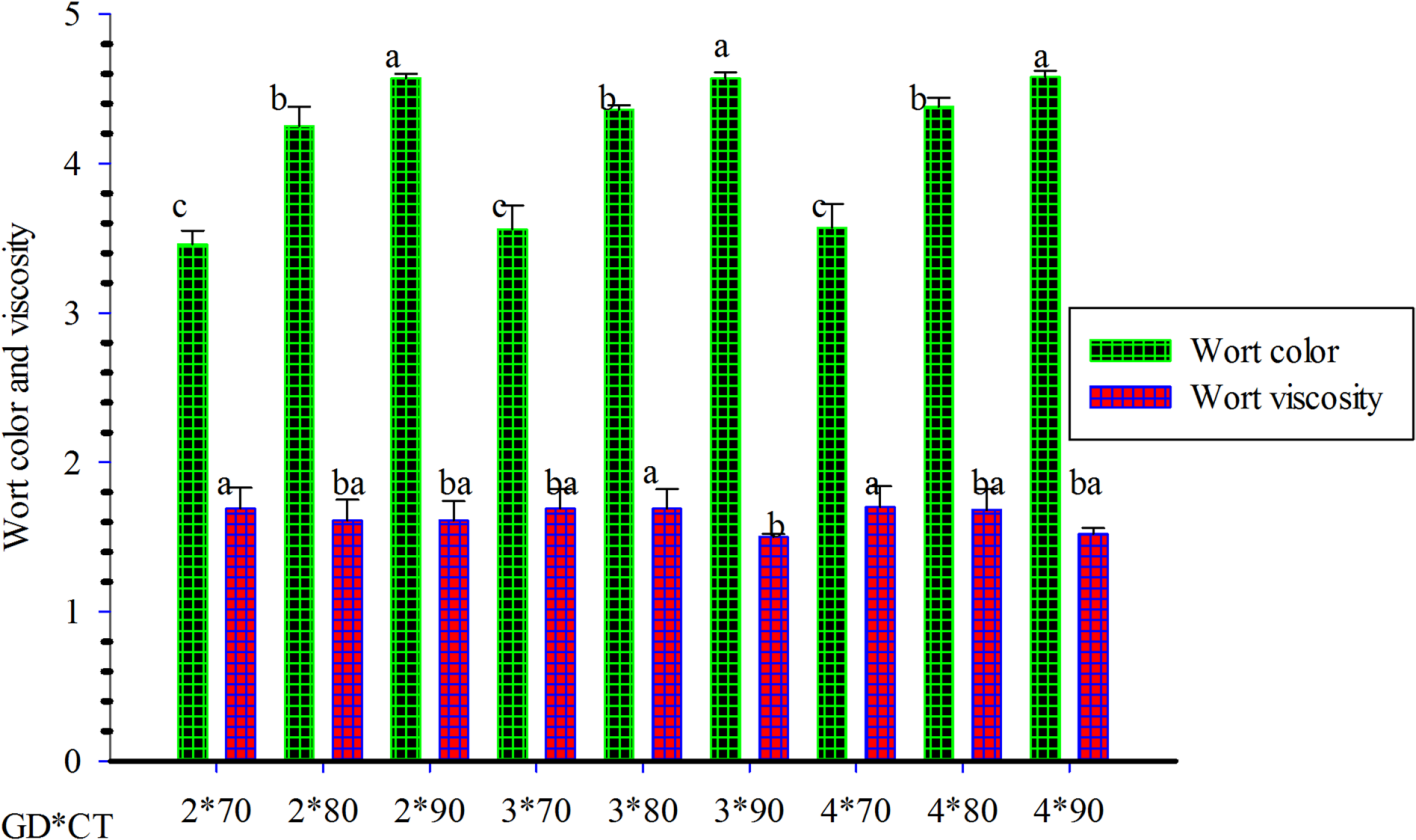
Effect of germination time and curing temperature on wort color and viscosity.

Wort color is one of the most important malt quality parameters in brewing^38^. Wort color increased from 4.06 to 4.24 as germination temperature rose from 20^*°*^C to 30^*°*^C. The color of the wort increased as curing temperature increased. This may be due to the browning reaction during the curing process. The result revealed significant difference (*p* < 0.05) between interactions for wort color. The wort color ranged from 3.35 to 4.63 EBC units shown in Fig. 3.

The lowest value (3.35) of wort color was shown at malt germinated at 20^*°*^C for 2 days and cured at 70^*°*^C whereas the maximum value (4.63) of wort color was generated at malt germinated at 30^*°*^C for four days and cured at 90^*°*^C (Table 4). In this finding, most of the combinations are in the range of the EBC standard for wort color, which is 2.50–4.50 EBC units but, some of the combinations gave higher values than EBC standard specification. This phenomenon likely results from elevated pigment concentrations in finger millet matrices. Wort color development primarily depends on kilned malt type and mashing process parameters^57^. Malt modification degree, particularly protein modification extent, represents another critical factor influencing color formation.

The Maillard reaction mechanism, based on amino acid-reducing sugar interactions, facilitate enhanced color development in well-modified malts containing elevated reducing sugar concentrations and soluble nitrogenous compounds. This biochemical process explains the relationship between malt modification quality and resulting wort coloration intensity (Table 4).

**Table 4:** Interaction effect GT, Gt, and CT on finger millet wort color, pH, and DP.

Factors			Parameters		DP (WK^o^)
GT	Gt	CT	Wort Color	Wort pH	
20	2	70	3.35 ± 0.000 l	5.87 ± 0.045^f^	53.01 ± ^bedc^
		80	4.06 ± 0.045f	5.96 ± 0.000^ef^	38.27 ± ^fgh^
		90	4.55 ± 0.000b	6.10 ± 0.000^ebdfc^	45.01 ± ^fgedc^
	3	70	3.43 ± 0.000k	5.91 ± 0.025^f^	30.78 ± ^h^
		80	4.33 ± 0.000e	6.45 ± 0.050^a^	42.75 ± ^fgeh^
		90	4.55 ± 0.000b	6.41 ± 0.000^a^	43.33 ± ^fgedh^
	4	70	3.45 ± 0.000jk	6.02 ± 0.000^ef^	39.33 ± ^fgh^
		80	4.33 ± 0.000e	6.43 ± 0.015^a^	44.46 ± ^fged^
		90	4.55 ± 0.000b	6.21 ± 0.000^ebdac^	44.46 ± ^fged^
25	2	70	3.47 ± 0.005^ji^	5.86 ± 0.000^f^	39.33 ± ^fgh^
		80	4.33 ± 0.005^e^	6.30 ± 0.005^bdac^	34.30 ± ^gh^
		90	4.55 ± 0.000^b^	6.02 ± 0.005^ef^	40.60 ± ^fgeh^
	3	70	3.48 ± 0.000^i^	5.93 ± 0.005^f^	38.78 ± ^fgh^
		80	4.34 ± 0.000^e^	6.36 ± 0.010^ba^	41.04 ± ^fgeh^
		90	4.55 ± 0.000^b^	6.31 ± 0.000^bdac^	35.91 ± ^gh^
	4	70	3.48 ± 0.000^i^	5.92 ± 0.055^f^	42.75 ± ^fgeh^
		80	4.34 ± 0.005^e^	6.36 ± 0.000^ba^	42.12 ± ^fgeh^
		90	4.56 ± 0.000^b^	6.32 ± 0.000^bac^	51.36 ± ^fbedc^
30	2	70	3.57 ± 0.005^h^	5.99 ± 0.000^ef^	53.01 ± ^bedc^
		80	4.35 ± 0.000^e^	6.31 ± 0.000^bdac^	53.01 ± ^bedc^
		90	4.61 ± 0.000^a^	6.43 ± 0.000^a^	37.62 ± ^gh^
	3	70	3.77 ± 0.005^g^	6.05 ± 0.050^edf^	56.43 ± ^bdc^
		80	4.41 ± 0.005^gd^	6.32 ± 0.000^bac^	34.20 ± ^gh^
		90	4.63 ± 0.000^a^	5.99 ± 0.025^ef^	63.27 ± ^ba^
	4	70	3.79 ± 0.000^g^	6.01 ± 0.000^ef^	70.07 ± ^a^
		80	4.47 ± 0.000^c^	6.32 ± 0.421^bac^	61.56 ± ^ba^
		90	4.63 ± 0.005^a^	6.06 ± 0.000^edfc^	58.14 ± ^bac^
Pv			< 0.0001	< 0.0001	< 0.0001
CV			0.22	1.36	9.21

Mean values not followed with the same letter in a column are significantly different at *P* < 0.05.

Abbreviation: GT = germination temperature; Gt = germination time; CT = curing temperature.

#### Wort pH

Wort pH represents a crucial quality parameter in brewing applications, directly affecting enzyme activity, microbial stability, and final product characteristics. Our pH values ranged from 5.86 to 6.45, falling to be within acceptable ranges for brewing applications^58^. The wort pH values were not significantly different among the germination temperature, but it was raised as the germination time increased. The wort pH values were significantly affected (*p* < 0.05) by interaction of germination temperature, germination time and curing temperature. The pH value of the wort sample ranged from 5.86 to 6.45. The maximum pH value (6.45) of wort was shown at malt germinated at 20^*°*^C for 3 days and cured at 80^*°*^C followed by pH value (6.43) in malt sample germinated at 30^*°*^C for 2 days and cured at 90^*°*^C, whereas the lowest pH value (5.86) of wort was generated at malt germinated at 25^*°*^C for 2 days and cured at 70^*°*^C (Table 4). The variation of the pH limits the growth of microorganisms, specifically, the growth of fermenting yeasts is influenced within the variation of pH^60^, but in this work, the pH of the wort was in the specified range. It was stated that over the pH range from 5 to 6.6, the photolytic activity of malt can vary^60^.

#### Diastatic power

Diastatic power is a measure of the proportion of available enzyme capable of converting complex starches into fermentable sugar (Ofoedu et al., 2022). Diastatic power was shown increment as both germination temperature and germination time increased. These results agree with those reported by^63^.

In the case of three-way interaction effect the maximum value (70.07 WK units) of diastatic power was recorded at malt germinated at 30^*°*^C for 4 days and cured at 70^°^C. On the other hand, the minimum value (30.78 WK units) was obtained at malt germinated at 20^*°*^C for 3 days and cured at 70^*°*^C (Table 4). The millet malt diastatic power is much lower than the diastatic power of barley malt^63^. The possible reason might be due to the lower protein content of finger millet than barley. The diastatic power value for finger millet was reported in the range of 34.7 to 45.1SDU/g^65^. According to^66^, diastatic power of 15 SDU/g was recorded as the highest value for finger millet. In this finding, the DP value was varied in the range of 30.78–70.07 WK units which is low than the acceptable levels of DP ranging from 110 to 150 SDU/g in typical commercial high-kilned (80 °C) malts pointed out by Burger and LaBerge (1985). According to Yousif & Evans, 2020^67^ high level of DP was required in brewing processes and was an important characteristic for estimating the quality of malt for beer production.

#### Wort viscosity

Wort viscosity analysis revealed significant treatment effects, with values ranging from 1.49 to 1.79 cP. The majority of treatment combinations produced viscosity values within EBC acceptable ranges (1.45–1.60 cP), indicating suitable processing characteristics for commercial brewing applications. These values are significantly (*p* < 0.05) higher than the wort viscosity value (1.60mp) recorded in malt sample germinated at 20^*°*^C. As indicated in Table 5 the interaction of germination temperature, germination time and curing temperature had significant effect (*p* < 0.05) on the wort viscosity. Majority of treatment combinations were significant among each other. The highest (1.79 cP) and lowest (1.49 cp.) values of wort viscosity were found in samples germinated at 30^*°*^C for three days and cured at 70^*°*^C and at 20^*°*^C for four days and cured at a temperature of 80^*°*^C, respectively. The lowest values (1.49cp) of wort viscosity obtained in this finding fulfill the EBC standard of viscosity which is from 1.45-1.45.60cP. According to H. Rani & Bhardwaj, 2021^39^low wort viscosity is desirable because high level of viscosity reduces the efficiency of breweries due to difficulty with filtration.

Viscosity is highly associated with wort β-glucan but also influenced by another important non-starch polysaccharide, arabinoxylan. All the interaction means are also in the acceptable range of the EBC standard. According to Lencha & Solomon, 2021^68^ the industrial specification of the wort viscosity is ranged from 1.55 to 1.65 cP which is in line with the majority of the current study. A work by Solgajová et al., 2022^69^ on wort viscosity has been reported to vary between 1.59 and 5.16 cP for a specific gravity of 1.030–1.100. Solgajová et al., 2022^70^found that arabinoxylan, β-glucans and dextrins all increased the viscosity of model solutions with the dextrins having the largest effect. Similarly^71^, reported viscous worts could result when high molecular weight, water-soluble β-glucans are degraded sufficiently during the malting process that could favour high molecular weight to be part of the wort on filtration.

Wort and beer viscosity significantly influences brewing processes and final product quality through multiple mechanisms. Beer viscosity contributes positively to mouthfeel and body characteristics. Elevated viscosity retards liquid drainage from foam bubble walls, enhancing head retention properties of beer foams.

However, excessive wort and beer viscosity reduces operational efficiency across numerous unit operations including mash stirring, pumping systems, wort-spent grain separation, wort boiling, thermal cooling, beer clarification processes, and filtration operations^71^. This dual nature of viscosity effects requires careful optimization to balance sensory attributes with processing efficiency.

**Table 5:** The interaction effect of GT, Gt and CT on Viscosity, HWE, and FAN.

Factors			Parameters		
GT(°C)	Gt (Day)	CT (°C)	Viscosity	HWE (%,db)	FAN
20	2	70	1.51 ± 0.01^b^	83.22 ± 0.95^i^	133.0 ± 0.00^a^
		80	1.51 ± 0.01^b^	89.20 ± 0.07^bc^	119.0 ± 1.00^c^
		90	1.51 ± 0.01^b^	77.67 ± 0.49^l^	118.5 ± 2.50^c^
	3	70	1.77 ± 0.02^a^	80.04 ± 0.58^kj^	132.0 ± 0.00^ab^
		80	1.78 ± 0.01^a^	85.53 ± 0.52^hg^	120 ± 1.00^c^
		90	1.51 ± 0.02^b^	84.70 ± 0.50^hi^	118.5 ± 2.50^c^
	4	70	1.78 ± 0.01^a^	80.61 ± 0.18^kj^	132.0 ± 0.00^ab^
		80	1.49 ± 0.01^b^	88.02 ± 0.00^edc^	120.5 ± 0.00^c^
		90	1.51 ± 0.01^b^	88.63 ± 0.50^bdc^	118.0 ± 0.50^c^
25	2	70	1.75 ± 0.08^a^	79.16 ± 0.49^kl^	131.00 ± 3.00^ab^
		80	1.78 ± 0.01^a^	87.10 ± 0.00^fe^	120.0 ± 0.50^c^
		90	1.77 ± 0.02^a^	79.99 ± 0.15^kj^	118 ± 1.00c
	3	70	1.51 ± 0.01^b^	80.27 ± 0.00^kj^	128 ± 3.00^ab^
		80	1.79 ± 0.01^a^	86.24 ± 1.00^fg^	119.5 ± 1.50^c^
		90	1.52 ± 0.01b	88.26 ± 1.00^bedc^	117 ± 2.50^c^
	4	70	1.51 ± 0.02^b^	90.72 ± 0.50^a^	127.0 ± 0.00^b^
		80	1.78 ± 0.02^a^	86.35 ± 0.00^fg^	119 ± 2.00c
		90	1.53 ± 0.05^b^	86.30 ± 0.00^fg^	117.5 ± 2.50^c^
30	2	70	1.78 ± 0.01^a^	89.76 ± 0.50^ba^	121.5 ± 0.50^c^
		80	1.51 ± 0.01^b^	89.15 ± 0.05^bc^	119.0 ± 2.0^c^
		90	1.56 ± 0.01^b^	87.49 ± 0.50^fed^	118 ± 1.00^c^
	3	70	1.79 ± 0.11^a^	81.15 ± 0.01^j^	121.5 ± 0.50^c^
		80	1.51 ± 0.01^b^	88.50 ± 0.50^bedc^	119.0 ± 2.00^c^
		90	1.51 ± 0.01^b^	87.49 ± 0.50^fed^	117.5 ± 1.50^c^
	4	70	1.78 ± 0.01^a^	81.14 ± 0.00^j^	121.0 ± 0.00^c^
		80	1.78 ± 0.02^a^	78.16 ± 0.00^l^	118.5 ± 2.50^c^
		90	1.53 ± 0.05^b^	89.49 ± 0.50^bac^	117.0 ± 2.00^c^
p-v			< 0.0001	< 0.0001	< 0.0001
cv			2.16	0.55	2.16

Mean values not followed with the same letter in a column are significantly different at *P* < 0.05.

Abbreviation: GT = germination temperature; Gt = germination time; CT = curing temperature.

#### Hot water extract (HWE)

Malt hot water extract is used to determine the amount of beer that can be produced from a known quantity of malt (Ofoedu et al., 2022). The HWE was significantly affected (*p* < 0.05) by germination temperature, germination time and curing temperature. The HWE content of the malt sample studied varied from 77.67% to 90.72%. The HWE(90.72%) was more prominent in malt sample germinated at 25 °C for four days and cured at a 70^*°*^C followed by the malt sample (89.76%) at 30^*°*^C for two days and cured at 70^*°*^C (Table 5). In this finding the malt sample germinated at 20^*°*^C for two days and cured at 90^*°*^C had the lowest HWE value (77.67) compared to the other treatments. This finding agreed with the finding of^73^ who had reported the hot water extract content of sorghum malt ranged from 71.1 to 85.2%. According to the quality standards of Asella Malt Factory, the minimum industrial specification of malt hot water extract is 76.00%. The means of EBC standard value ranges from 79.00 to 82.00%. The hot water extrat content resulted in this study fulfilling the standards set by Asella Malt Factory. But the highest HWE value (90.72) obtained in this work is higher than the maximum specification of EBC standards, 82%. According to^74^ the qulity of the extract is influenced by several factors. One of the most important factors that influences extract is the malting processes^61^. Malt extract is also dependent on the grain germination capacity and catabolic activities mediated by amylase, protease, and glucanase activities. Because of high glucan content in grain, ungerminated and incompletely modified seeds cause low malt extract, slow filtration rate and high molecular nitrogen in the extract, which results in low quality of beer^74^.

#### Free amino nitrogen (FAN)

During the fermentation process, sufficient amount of FAN is used by yeasts for building protein-supporting biomass growth. In addition, it is partially used for creating volatile compounds, including higher alcohols, esters, diacetyl and H_2_S^75^.

The FAN content decreased as the curing temperature increased as stated in Table 5. The analysis result revealed that the interaction between factors was significantly different (*p* < 0.05) for free amino nitrogen. In this study, the FAN content ranged from 117 mL/L to 133 mL/L (Table 5). The malt germinated at 20^*°*^C for 2 days and cured at 70^*°*^C exhibited the highest FAN content (133 mL/L), whereas the malt sample germinated at 30^*°*^C for 4 days and cure at a temperature of 90^*°*^C had the lowest FAN content (117 mL/L). Because the higher germination temperature and curing temperature leads to higher browning reaction which reduces FAN, since during the browning reaction the free amino nitrogen reacts with simple sugar of the malt.

According to Shayo et al., 2001^65^ report, the value for FAN in finger millet ranged from 87 to 155 ml/L. On other hand 118 ml/L was recorded as the highest free amino nitrogen values for finger millet as reported^76^. Adequate levels of FAN (around 130 mg/L in wort ensure efficient and optimum yeast cell growth and, hence, a desirable fermentation performance^77^. According to Djameh et al., 2015^78^ optimization of the sorghum free amino nitrogen increased as germination time increased from three to five days. Wort free amino nitrogen increased with germination time in the experimental malt, indicating higher modification in the kernels. But in current finding higher FAN was obtained at lower germination time due to impact of germination temperature and curing temperature in case of three-way interaction. Generally, the desirable quality specifications for a normal fermentation require FAN levels between 140 and 160 mg/L which are somehow higher than the current finding. Fouquet et al., 2022^79^ also describe the malting industry specifications to range from 140 to 180 mg/L for efficient fermentation. According to^42^ the FAN levels of the wort is largely dependent on the malt or the grist used, whilst the mashing program has only a limited influence.

## Conclusion

This comprehensive study demonstrates that finger millet (Mecha variety) possesses excellent malting characteristics and significant potential for commercial beer production. The research successfully established optimal malting conditions, with germination at 30 °C for 4 days achieving the highest germination energy of 97.66%, exceeding international quality standards for brewing grains. The findings reveal that malting process parameters significantly influence both malt and wort quality attributes. Higher germination temperatures and extended germination periods generally enhanced enzyme development and grain modification, though careful balance is required to minimize excessive weight losses. The hot water extract values ranging from 77.67% to 90.72% demonstrate that finger millet malt meets industrial brewing specifications, with some treatments surpassing EBC standards. Particularly noteworthy is finger millet’s superior foam stability compared to conventional brewing materials, attributed to its natural tannin content. The varied diastatic power values (30.78–70.07 WK units) indicate adequate enzymatic activity for starch conversion, though supplementation strategies may optimize brewing efficiency. The study establishes finger millet as a viable alternative to barley malt, offering brewers opportunities for product diversification while supporting agricultural sustainability. Study limitations include the laboratory-scale experimental conditions, focus on a single finger millet variety, and absence of microbial stability analysis or sensory evaluation. Future research priorities should include pilot-scale brewing trials to validate laboratory findings, comprehensive sensory evaluation of finger millet-based beers, economic feasibility assessments for commercial implementation, and investigation of enzyme supplementation strategies to optimize diastatic power limitations.

## Supplementary Information

Below is the link to the electronic supplementary material.

Supplementary Material 1

## Data Availability

The data supporting the findings of this study are available from the corresponding author, Reddy Prasad D.M. (dmr.prasad@utb.edu.bn), upon reasonable request.
