# Supplementary material for: Impact of germination and kilning parameters on Eleusine coracana malting for industrial brewing applications
Source: Sci Rep. 2025 Dec 29;15:44774. doi: 10.1038/s41598-025-28926-2 (PMC12749404; doi:10.1038/s41598-025-28926-2)
Supplement: Supplementary file 1 — Supplementary Material 1 [file 41598_2025_28926_MOESM1_ESM.docx]

**Appendix -A**

**Quality Standards Reference Table for Malt and Brewing Parameters**

Table1 compares barley and malt quality standards across different organizations including EBC Standard, EQSA (Ethiopia), Asella Malt Factory, and Dashen Brewery, covering both grain quality metrics (germination energy, hectoliter weight) and malt quality parameters. The table provides specific values for critical brewing parameters like hot water extract, moisture content, wort viscosity, and protein content, along with explanatory notes for each parameter's significance in the malting process.

**Table 1: Comprehensive Quality Standards Comparison**

| **Parameter** | **Unit** | **EBC Standard** | **EQSA (Ethiopia)** | **Asella Malt Factory** | **Dashen Brewery** | **Notes** |
| --- | --- | --- | --- | --- | --- | --- |
| **GRAIN QUALITY** | | | | | | |
| Germination Energy (72h) | % | - | Grade 1: 95%<br>Grade 2: 92%<br>Grade 3: 90% | - | - | Minimum 90% required for malting |
| Hectoliter Weight | kg/hL | 65-75 | Grade 1: 65<br>Grade 2: 62<br>Grade 3: 60 | - | Grade 1: 75<br>Grade 2: 70<br>Grade 3: 68 | Higher values indicate better quality |
| **MALT QUALITY** | | | | | | |
| Moisture Content | % | 3.0-5.8 | - | ≤5.8 | - | Critical for storage stability |
| Total Protein | % | ≥9.0 | - | - | - | Minimum for adequate nutrition |
| Kolbach Index | % | Under-modified: <35<br>Well-modified: 35-41<br>Over-modified: 41-43 | - | - | - | Protein modification indicator |
| **WORT QUALITY** | | | | | | |
| Wort Color | EBC units | 2.5-4.0 | - | - | - | Lighter colors preferred |
| Wort pH | - | 5.0-6.6 | - | - | - | Affects enzyme activity |
| Viscosity | cP | 1.45-1.60 | - | - | - | Lower values improve processing |
| Hot Water Extract | % (db) | 79.0-82.0 | - | ≥76.0 | - | Extract yield indicator |
| Free Amino Nitrogen | mg/L | 140-180 | - | - | - | Required for yeast nutrition |
| Diastatic Power | WK units | 110-150 | - | - | - | Enzyme activity measure |
| Calcium Content | mg/L | - | - | - | - | Essential for brewing chemistry |

**Table 2: Grade Classifications: EQSA (Ethiopian Quality Standards Authority) Barley Malt Grades**

Table2 presents Ethiopia's official barley grading classification system with three distinct grades based on two key quality indicators: germination energy and hectoliter weight.

| **Grade** | **Germination Energy (%)** | **Hectoliter Weight (kg/hL)** |
| --- | --- | --- |
| 1st Grade | 95 | 65 |
| 2nd Grade | 92 | 62 |
| 3rd Grade | 90 | 60 |

**Table 3: Acceptable Quality Ranges for Key Malting Parameters**

Table 3 defines the minimum acceptable, optimal range, and maximum acceptable values for essential malting parameters including germination energy, moisture content, hot water extract, wort viscosity, and protein content.

| **Parameter** | **Minimum Acceptable** | **Optimal Range** | **Maximum Acceptable** |
| --- | --- | --- | --- |
| Germination Energy | 90% | 95-98% | - |
| Moisture Content | - | 3-5% | 5.80% |
| Hot Water Extract | 76% | 79-82% | - |
| Wort Viscosity | - | 1.45-1.60 cP | 1.60 cP |
| Free Amino Nitrogen | 130 mg/L | 140-160 mg/L | 180 mg/L |
| Diastatic Power | 30 WK | 110-150 WK | - |

**References for Standards:**

- **EBC**: European Brewery Convention Standards
- **EQSA**: Ethiopia Quality Standards Authority (ES 673:2023)
- **Asella Malt Factory**: Industrial specifications for Ethiopian malt production
- **Dashen Brewery**: Commercial brewery requirements in Ethiopia
